# From red to green: the propidium iodide-permeable membrane of *Shewanella decolorationis* S12 is repairable

**DOI:** 10.1038/srep18583

**Published:** 2015-12-21

**Authors:** Yonggang Yang, Yinbo Xiang, Meiying Xu

**Affiliations:** 1 Guangdong Provincial Key Laboratory of Microbial Culture Collection and Application, Guangdong Institute of Microbiology, Guangzhou, China; 2 State Key Laboratory of Applied Microbiology Southern China, Guangzhou, China

## Abstract

Viability is a common issue of concern in almost all microbial processes. Fluorescence-based assays are extensively used in microbial viability assessment, especially for mixed-species samples or biofilms. Propidium iodide (PI) is the most frequently used fluorescence indicator for cell viability based on the membrane permeability. Our results showed that the accumulation of succinate from fumarate respiration could induce PI-permeability in *Shewanella decolorationis* biofilm cells. Confocal laser scanning microscope further showed that the PI-permeable membrane could be repaired *in situ* when the extracellular succinate was eliminated by switching fumarate respiration to electrode respiration. Simultaneously with the membrane repair, the electrode respiring capacity of the originally PI-permeable cells was recovered. Agar-colony counts suggested that a major portion of the repaired cells were viable but nonculturable (VBNC). The results evidenced that *S. decolorationis* S12 has the capacity to repair PI-permeable membranes which suggests a reevaluation of the fate and function of the PI-permeable bacteria and expanded our knowledge on the flexibility of bacterial survival status in harsh environments.

Microbial viability is a common concern in almost all microorganism-involved natural or artificial systems (e.g. bioremediation, biosynthesis or infection). Counting colony forming units (CFU) on agar plates is considered a “gold standard” in microbial viability assessment^1^,^2^, but is often unsuitable for complex samples such as mixed-species communities, biofilms or environmental samples. Moreover, the culturability presented by CFU cannot provide real-time and specific status of microorganisms in a bioprocess considering the plate-growing procedure and that microorganisms have many intermediate status (e.g. deenergized or depolarized cells)^3^,^4^,^5^.

Fluorescence staining integrating with microscopic techniques have been increasingly used, especially for various environmental samples containing unknown or nonculturable microbes^5^. Membrane integrity is essential for living cells and therefore is regularly used as a viability indicator. Propidium iodide (PI) has been used in numerous reports to distinguish the dead or nonviable cells from living prokaryotic or eucaryotic cells, as PI was believed to only stain cells with irreparably damaged membranes that can be described as nonviable or dead^1^,^5^,^6^,^7^. However, it was recently evidenced that *Saccharomyces cerevisiae* can repair the stress-induced PI-permeable membrane by delimiting chemical stresses^1^. Whether bacterial cells have a similar capacity is yet unknown, despite the methodological and physiological importance of this question.

Biofilms are the general living form of bacteria in natural and engineered environments^6^. *Shewanella decolorationis* S12 is a biofilm-forming strain that was isolated from sludge in a wastewater treatment plant^8^. This strain has multiple respiration strategies using electron acceptors such as mineral oxides, nitrate, azo compounds, electrodes or fumarate and a strong adaptability to contaminated environments^8^,^9^. In this study, we provide evidence that *S. decolorationis* S12 biofilm cells are able to repair PI-permeable membranes, which advances bacterial membrane flexibility as a possible strategy for survival in harsh environments.

## Results and Discussion

### PI-permeable *S. decolorationis* cells grown with fumarate

**Figure 1:**
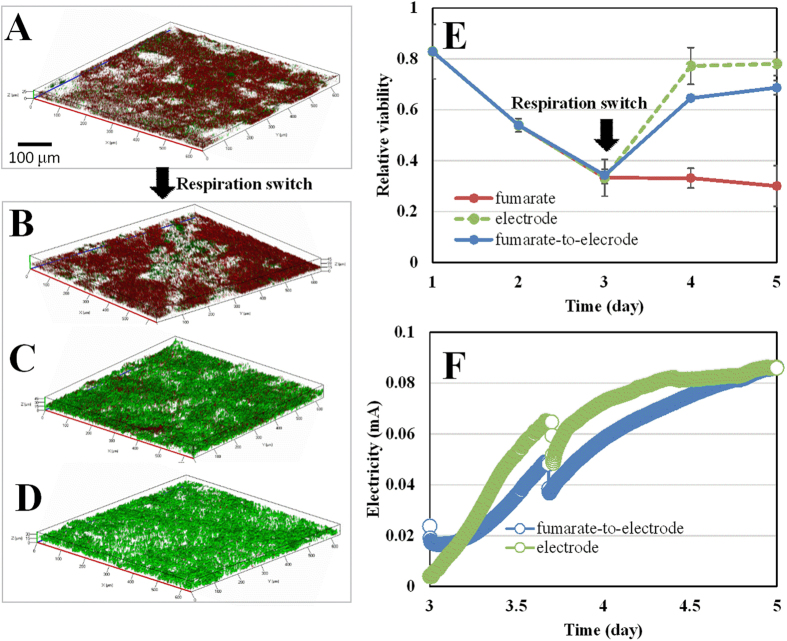
The repairability of the PI-permeable S. decolorationis S12 biofilm cells with simultaneous recovery of electrode respiring capacity. (**A**) 3-day old fumarate-grown biofilm before respiration-siwtch; (**B**) 5-day old biofilm maintained with fumrate as the sole electron acceptor; (**C**) 5-day old biofilm after switching from fumarate- to electrode-respiration; (**D**) 2-day old electrode-grown biofilm in a new BER; (**E**) time-course of PI-based relative viability of different biofilms before and after respiration switch (indicated by arrow); (**F**) electricity generation of the fumarate-to-electrode biofilms in comparison with the newly electrode-grown biofilm in the newly assemmbled BER.

Firstly, we grew *S. decolorationis* S12 biofilms on graphite plates in two identical bioelectrochemical reactors (BERs) wherein fumarate was provided as the sole electron acceptor. After 72 h, rough biofilms with an even thickness of 26 μm formed on the graphite plates (Fig. 1), and the protein-based biofilm biomass increased to 37.7 μg/cm^2^ (Fig. S1). However, the proportion of cells with PI-permeable membranes (red) gradually increased from the first day and the relative biofilm viability (the ratio of green to total cells in the biofilms) decreased to 0.33 after 72 h, much lower than that of the *Shewanella* biofilms grown with several other electron acceptors (≥0.7)^9^,^10^,^11^.This is in line with previous reports that a high concentration of fumarate (≥40 mM) could inhibit the growth of *S. oneidensis* MR-1^12^ and that fumarate-grown *Geobacter sulfurreducens* biofilm cells showed obviously higher PI-permeability compared with electrode-grown cells^13^. The viability loss of fumarate-grown biofilms can be understood based on the recent finding that extracellular accumulation of succinate from fumarate-reduction can impair bacterial membrane and decrease cell viability^14^. In line with that, when we increased the fumarate concentration from 5 to 40 mM (Fig. S2) the biofilm viability decreased, despite more energy could be obtained by respiring with higher concentration of fuamrte. The results suggest that the viability decrease was caused by succinate accumulation from fumarate reduction, rather than starvation.

### The repairablity of the PI-permeable membrane of *S. decolorationis* cells

After the first fumarate-respiration period, one of the reactors was switched to electrode respiration (fumrate-to-electrode reactor), one was maintained as fumarate-respiration (fumarate reactor) and the third one was operated with a biofilm-free anode and oxygen-grown strain S12 as a new electrode-respiring reactor. Upon respiration-switch, the fumarate-to-electrode and electrode reactors showed immediate electricity generation with different initial electricity levels (17 and 5 μA respectively, Fig. 1F). The biofilm biomass in the fumarate-to-electrode and electrode reactors dramatically increased (by 14.7 and 37.4 μg/cm^2^ respectively, Fig. S1B). In contrast, a slight increase occurred in the fumarate-maintained reactor because some of the planktonic cells attached to the anode surface to facilitate electrode respiration. This is in line with the proportional decrease in planktonic cell densities (Fig. S1B). Planktonic *Shewanella* cells “swimming” towards an electrode has been demonstrated as an energy taxis process driven by the redox-gradient of electron shuttles, i.e. flavins^15^. Despite different initial electricity levels, the fumarate-to-electrode and electrode reactors showed comparable maximum electricity levels (83 *vs* 87 μA) and electricity generation capacities (0.16 *vs* 0.18 μA per μg protein, normalized to the biofilm protein content) after a 48-hour adaption period. Biofilms play a dominant role in *S. decolorationis* electricity generation^16^,^17^. Therefore, the results indicate a recovery of the electrode-respiring capacity of the PI-permeable biofilm cells.

After the respiration-switch, biofilm viability in the fumarate reactor showed a further decrease from 0.33 to 0.3 (Fig. 1E). In contrast, the viability of the fumarate-to-electrode grown biofilms increased from 0.33 to 0.71 while the viability of the newly assembled electrode-grown biofilms was 0.78. The observed viability recovery of the fumarate-to-electrode grown biofilm could be attributed to two possible reasons: (i) the PI-permeable membranes of the biofilm cells were repaired, or (ii) new intact cells were generated in the biofilms. Considering that the biomass in the fumarate-to-electrode biofilm increased by only 12.5% (Fig. S1B) after respiration switch which could not solely account for the dramatic increase (by 115%) in biofilm viability. Therefore, it can be deduced that the *in situ* viability recovery of the fumarate-to-electrode biofilms were primarily caused by repair of the PI-permeable cell membrane.

It has been reported that PI-permeable cells of several bacterial organisms including *Mycobacterium frederiksbergense*, *Sphingomonas* sp. LB126, *S. oneidensis* MR-1 and two *Bifidobacterium lactis* strains, are able to actively grow or express proteins at a comparable level to cells with intact membranes^4^,^18^,^19^. In addition to the viability maintained by these PI-labeled bacteria, our results further evidenced an *in situ* repairablity of PI-permeable membranes by bacterial cells with simultaneous recovery of energy metabolism (electrode-respiring) activity.

### The culturablility of the repaired biofilm cells

**Figure 2:**
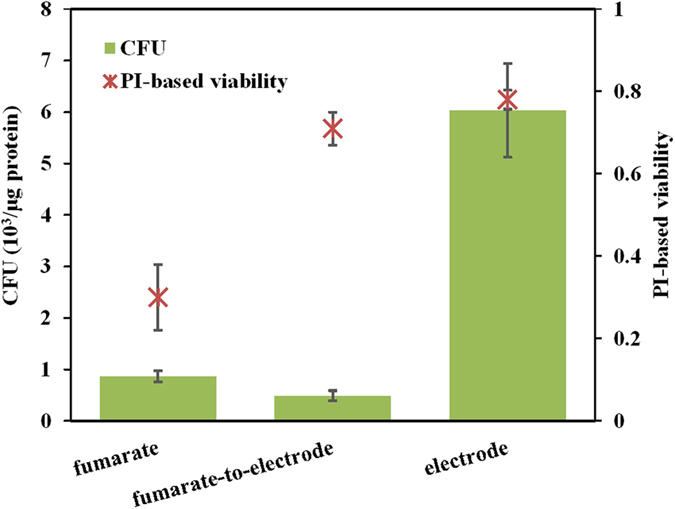
Comparison of the CFU and PI-based viability of biofilm cells after respiration switch.

Agar-plate based CFU count, as a classical bacterial culturablility assay, was performed to verify the reliability of the PI-based biofilm viability. However, the two assays showed inconsistent results. Specifically, the fumarate-to-electrode grown biofilm showed a comparable PI-based viability with the “green” electrode-grown biofilm but a comparable culturablility with the “red” fumarate-grown biofilm (Fig. 2), indicating that a major portion of the membrane-repaired cells were still unculturable on the aerobic Luria-Bertani plate. Bacterial cells with intact membranes have various transient statuses which might be inconsistent with the culturablility presented by the CFU assay^3^,^7^. Those viable but nonculturable (VBNC) cells have been recognized as an important physiological state for bacterial survival in harsh environments^2^. Many factors, such as starvation, temperature or osmotic concentration, may induce VBNC, and the PI-impermeable membrane is an important characteristic to distinguish VBNC cells from dead cells^2^. Although electrode-respiration may elevate the stress level of *Shewanella* cells^20^, succinate accumulation was most likely the reason for the VBNC cells as the biofilm cells in the newly assembled electrode reactor showed high viability and culturability (Fig. 2).

In summary, our results showed that the accumulation of extracellular succinate in fumarate respiration induced PI-permeable cell membranes of *S. decolorationis* S12 which could be repaired to transient VBNC status with intact cell membrane by eliminating the succinate stress by switching fumarate-respiration to electrode-respiration. PI-permeability is among the most commonly used methods to assess the viability of microorganisms. Our results highlighted the fact that *S. decolorationis* S12 have the capacity of repairing the PI-permeable cell membrane and recovering metabolic activity. Therefore, it is unreliable to PI-permeability as the only method in determining bacteria viability, some other physiological analyses should be combined. Moreover, the reversible membrane permeability is possibly a survival or evolution strategy of some microbes (such as *Shewanella decolorationis*) exposed to harsh environments, as the permeable membranes can conserve energy and increase physical and chemical communication between microbial cells and surrounding environment.

## Materials and Methods

### Bacterial strain and grown conditions

*S. decolorationis* strain S12^T^ (CCTCC M203093^T^ = IAM15094^T^) was isolated from activated sludge at a textile-printing wastewater treatment plant and preserved in our laboratory^8^. A single colony of *S. decolorationis* S12 from an LB plate was inoculated into sterilized LB broth overnight at 30 °C. The resulting cells were separated from the culture by centrifugation (6000 × g) for 2 min, and washed twice using sterilized phosphate buffer solution (PBS, pH7.4) containing 137 mM NaCl, 2.7 mM KCl, 10 mM Na_2_HPO_4_ and 2 mM KH_2_PO_4_ to remove residual nutrients for bioreactor inoculation.

Three bioelectrochemical reactors (BER) were assembled as previously described^10^. The anode chamber of each BER contained 100 mL of anaerobic limited mineral medium (12.8 g/L Na_2_HPO_4_, 3 g/L KH_2_PO_4_, 0.5 g/L NaCl, 1.0 g/L NH_4_Cl and 0.05% (w/v) yeast extract) with 10 mM lactate (electron donor) and 20 mM of fumarate (electron acceptor) and was inoculated with the aerobic grown *S. decolorationis* cells. For the first three days, the anode and cathode of the three BES were disconnected to allow fumarate to serve as the sole electron acceptor for *S. decolorationis* biofilm growth. After that, the media in the BES was transferred to a (container) and centrifuged (rpm, time) to collect the planktonic cells. The BES were then restored by suspending the planktonic cells in fresh medium (as described below) and returning them to the corresponding BES (anode biofilms were undisturbed unless indicated): (i) fumarate respiration condition: supplement anode chamber with fresh LM medium with 10 mM of lactate and 20 mM fumarate to maintain fumarate respiration; (ii) fumarate-to-electrode respiration condition: supplement anode chamber with 100 ml fresh lactate-containing and fumarate-free LM medium; (iii) electrode-respiration condition: replace the anode biofilm and planktonic culture with a new biofilm-free electrode and fresh lactate-containing, fumarate-free LM medium, and inoculate the anode chamber with newly aerobic grown *S. decolorationis* S12 cells to a planktonic density equal to that of the other two reactors. The anode and cathode of the fumarate-to-electrode and electrode respiration reactors were then connected to initiate electrode-respiration (Fig. 1C). The current (i.e. electrode respiration rate) between the anode and cathode of the reactors with 1000 ohm resistors was monitored with a multimeter (Keithley 2700, module 7702).

### Biofilm viability assay and CFU count

A confocal laser scanning microscope (CLSM, LSM 700, Zeiss) was used to analyze the biofilm structure and viability. Before CLSM analysis, a piece of graphite plate with biofilm (0.5 × 1 cm) was sampled from each reactor in an anaerobic glove box and was dipped in sterilized PBS to remove loosely attached planktonic cells or debris. The sample was then stained with LIVE/DEAD BacLight staining kit (Molecular Probes, Invitrogen), by which cells with damaged membrane can be stained by PI and visualized as red cells while the intact cells are stained by both PI and SYTO 9 and visualized as green cells. Randomly sampled view-fields of each biofilm were observed (n ≥ 10), pixel-based biofilm viability was analyzed and presented as described before^9^,^10^. A CFU count was also performed with LB agar plates. The biofilm cells were scraped into LB broth with a sterilized blade. The LB broth containing biofilm cells was then gently blended to scatter the cell clusters and observed under a microscope to avoid cell death or clustering. The blended samples were spread on the LB agar plate and the CFUs were counted after 24 and 48 hours.

## Additional Information

**How to cite this article**: Yang, Y. *et al.* From red to green: the propidium iodide-permeable membrane of *Shewanella decolorationis* S12 is repairable. *Sci. Rep.* **5**, 18583; doi: 10.1038/srep18583 (2015).

## Supplementary Material

Supplementary Information
