# Supplementary material for: From red to green: the propidium iodide-permeable membrane of Shewanella decolorationis S12 is repairable
Source: Sci Rep. 2015 Dec 21;5:18583. doi: 10.1038/srep18583 (PMC4685271; doi:10.1038/srep18583)
Supplement: Supplementary Information [file srep18583-s1.pdf]

Supplementary information for

From red to green: the Propidium iodide-permeable membrane of *Shewanella decolorationis* S12 is repairable

Yonggang Yang[1, 2], Yinbo Xiang[2], Meiying Xu[1, 2*]

[1]Guangdong Provincial Key Laboratory of Microbial Culture Collection and Application, Guangdong Institute of Microbiology, Guangzhou, China

[2]State Key Laboratory of Applied MicrobiologySouthern China, Guangzhou, China

* Corresponding author, Guangdong Institute of Microbiology, 100# Xianlie middle road, Guangzhou, China 510070, Tel: +86 20 87684471, E-mail: xumy@gdim.cn.

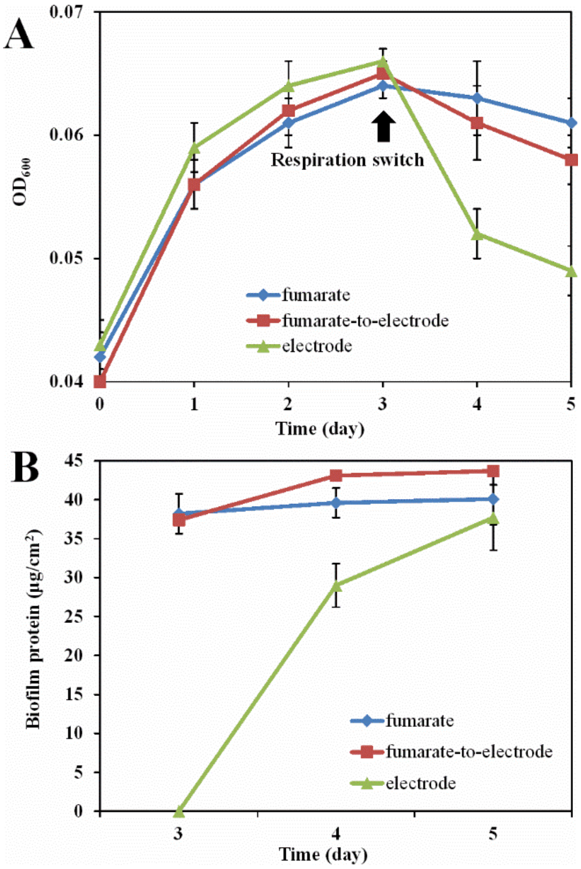

Fig. S1 The planktonic and biofilm cell growth before and after respiration switch.

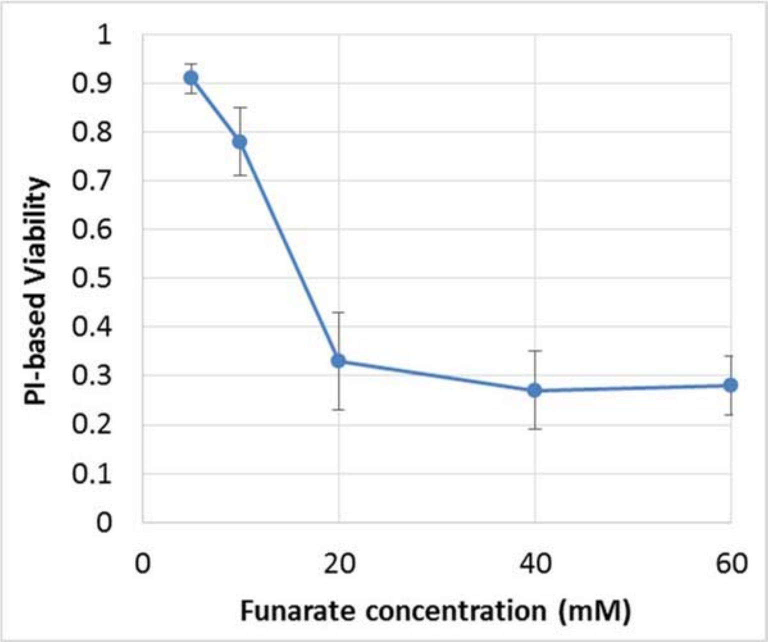

Fig.S2 Biofilm viability decreased with the fumarate concentration.
